# Comparison of model fit and discriminatory ability of M category as defined by the 7th and 8th editions of the tumor‐node‐metastasis classification of colorectal cancer and the 9th edition of the Japanese classification

**DOI:** 10.1002/cam4.3972

**Published:** 2021-09-29

**Authors:** Dai Shida, Narikazu Boku, Yuya Nakamura, Takefumi Yoshida, Taro Tanabe, Kohei Yasui, Atsuo Takashima, Yukihide Kanemitsu

**Affiliations:** ^1^ Department of Colorectal Surgery National Cancer Center Hospital Tokyo Japan; ^2^ Division of Frontier Surgery The Institute of Medical Science The University of Tokyo Tokyo Japan; ^3^ Gastrointestinal Medical Oncology Division National Cancer Center Hospital Tokyo Japan

**Keywords:** anal carcinoma, appendiceal, colorectal cancer, japanese Classification of colorectal, TNM 7th edition, TNM 8th edition

## Abstract

**Background:**

In transitioning from the 7th edition of the tumor‐node‐metastasis classification (TNM‐7) to the 8th edition (TNM‐8), colorectal cancer with peritoneal metastasis was newly categorized as M1c. In the 9th edition of the Japanese Classification of colorectal, appendiceal, and anal carcinoma (JPC‐9), M1c is further subdivided into M1c1 (without other organ involvement) and M1c2 (with other organ involvement). This study aimed to compare the model fit and discriminatory ability of the M category of these three classification systems, as no study to date has made this comparison.

**Methods:**

The study population consisted of stage IV colorectal cancer patients who were referred to the National Cancer Center Hospital from 2000 to 2017. The Akaike information criterion (AIC), Harrell's concordance index (C‐index), and time‐dependent receiver operating characteristic (ROC) curves were used to compare the three classification systems. Subgroup analyses, stratified by initial treatment year, were also performed.

**Results:**

According to TNM‐8, 670 (55%) patients had M1a, 273 (22%) had M1b, and 279 (23%) had M1c (87 M1c1 and 192 M1c2 using JPC‐9) tumors. Among the three classification systems, JPC‐9 had the lowest AIC value (JPC‐9: 10546.3; TNM‐7: 10555.9; TNM‐8: 10585.5), highest C‐index (JPC‐9: 0.608; TNM‐7: 0.598; TNM‐8: 0.599), and superior time‐dependent ROC curves throughout the observation period. Subgroup analyses were consistent with these results.

**Conclusions:**

While the revised M category definition did not improve model fit and discriminatory ability from TNM‐7 to TNM‐8, further subdivision of M1c in JPC‐9 improved these parameters. These results support further revisions to M1 subcategories in future editions of the TNM classification system.

## Graphical abstract

In transitioning from the TNM 7th edition to the TNM 8th edition, colorectal cancer with peritoneal metastasis was newly categorized as M1c, and in the Japanese Classification (9th edition), M1c is further subdivided into M1c1 (without other organ involvement) and M1c2 (with other organ involvement). To date, no study has compared the model fit and discriminatory ability of the M category of these three classification systems. In this study, with 1222 consecutive stage IV colorectal cancer patients, we clearly demonstrated that, by comparing AIC, Harrell's c‐index, and time‐dependent ROC curves between the three classification systems, while the revised M category definition did not improve model fit and discriminatory ability from TNM 7th edition to the TNM 8th edition, further subdivision of M1c in the Japanese Classification improved these parameters.

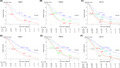

## 1 INTRODUCTION

Since the number of metastatic sites involved is an important prognostic factor for colorectal cancer,^1^ the 7th edition of the tumor‐node‐metastasis classification (TNM‐7) of malignant tumors (published in 2009)^2^ subdivides M1 into M1a and M1b: metastasis confined to one organ (liver, lung, ovary, or non‐regional lymph node(s)) is classified as M1a, and metastasis to more than one organ or the peritoneum as M1b. In the 8th edition of TNM (TNM‐8; published in 2017),^3^ colorectal cancer with peritoneal metastasis was categorized as M1c regardless of other organ involvement, which is experienced by approximately one fourth of patients presenting with M1 disease,^4^, ^5^ because its prognosis is worse than that for visceral metastases to one or more solid organs.^6^, ^7^ It is noteworthy that two studies^6^, ^7^ referred to in the American Joint Committee on Cancer (AJCC) cancer staging manual (8th edition)^8^ did not include as subjects all stage IV colorectal cancer patients, as those studies excluded patients with peritoneal metastasis who underwent curative resection.

Japan has its own classification system for colorectal cancer. The 9th edition of the Japanese Classification of colorectal, appendiceal, and anal carcinoma (JPC‐9) was published in 2018 by the Japanese Society for Cancer of the Colon and Rectum.^9^ In JPC‐9, M1c is further subdivided into M1c1 (metastasis to the peritoneum without other organ involvement) and M1c2 (metastasis to the peritoneum with other organ involvement).^10^

To date, no study to our knowledge has compared TNM‐7, TNM‐8, and JPC‐9 in detail. Thus, comparing these three classification systems may be informative. To this end, the present study aimed to compare the model fit and discriminatory ability of these three classification systems. In addition, an important consideration when evaluating long‐term outcomes is the use of a classification system which holds up when applied to both the past and present,^2^, ^3^ particularly in view of advances in diagnostic and treatment modalities which have improved the overall survival (OS) of stage IV colorectal cancer patients. This aspect was examined by performing subgroup analyses, in which patients were divided into two groups by initial treatment year.

## 2 MATERIALS and METHODS

### 2.1 Study population

Subjects were stage IV colorectal cancer patients who were referred to the Department of Colorectal Surgery or the Gastrointestinal Medical Oncology Division of the National Cancer Center Hospital from January 2000 to December 2017. Patients with appendiceal cancer or anal cancer, those with a histologic diagnosis other than adenocarcinoma (e.g., neuroendocrine carcinoma), and those with other concomitant advanced disease were excluded. The initial treatment strategy, such as curative resection including metastasectomy, palliative resection, and perioperative and palliative chemotherapy, was routinely decided during multidisciplinary team meetings attended by colorectal surgeons, medical oncologists, hepatobiliary surgeons, thoracic surgeons, and radiologists, taking into consideration disease severity, comorbidities, and patient condition.

This retrospective study was approved by the Institutional Review Board (IRB) of the National Cancer Center Hospital (IRB code: 2015–320).

### 2.2 Data collection

The following parameters were retrospectively assessed using medical records: treatment year, gender, age, Eastern Cooperative Oncology Group (ECOG) performance status (PS) at initial treatment, primary tumor site (right‐sided: cecum, ascending colon, hepatic flexure, and transverse colon; left‐sided: splenic flexure, descending colon, sigmoid colon, rectosigmoid junction, and rectum^11^), histological differentiation, type of systemic chemotherapy regimen (cytotoxic agent therapy without molecular targeted agents, such as fluoropyrimidine monotherapy, fluoropyrimidine plus oxaliplatin, and fluoropyrimidine plus irinotecan), use of at least one molecular targeted agent throughout the treatment course (i.e., bevacizumab, cetuximab, or panitumumab), and type of surgery (i.e., curative resection achieving R0 such as primary tumor resection and metastasectomy, including dissection of peritoneal metastasis; palliative resection such as primary tumor resection without metastasectomy; and unresected cases, including surgical procedures such as diverting stoma construction, bypass surgery, or probe laparotomy).

Information on the status of distant metastases was also collected and patients were categorized into two M subcategories (M1a, M1b) according to TNM‐7,^2^ three M subcategories (M1a, M1b, M1c) according to TNM‐8,^3^ and four M subcategories (M1a, M1b, M1c1, M1c2) according to JPC‐9.^9^

### 2.3 Treatment year subgroup analyses

Molecular targeted agents (bevacizumab, cetuximab, and panitumumab) were approved for treating stage IV colorectal cancer in Japan after 2007 (bevacizumab in 2007, cetuximab in 2008, and panitumumab in 2010). Thus, patients were stratified by initial treatment year for subgroup analyses (either 2000–2007 or 2008–2017).

### 2.4 Statistical analysis

Pearson's chi‐square test for categorical variables and the Wilcoxon rank‐sum test for continuous variables were performed to compare various patient background factors between the two subgroups (2000–2007 and 2008–2017). OS was defined as the interval between the date of stage IV colorectal cancer diagnosis and the date of death from all causes. Patients alive at the end of follow‐up (March 31, 2020) were censored. Kaplan–Meier plots were used to estimate OS. Differences in survival were assessed with the log‐rank test. The Akaike information criterion (AIC) is an information‐based criterion that assesses model fit and can be used to compare various models with the same data set. AIC was calculated as follows: AIC = −2 log maximum likelihood +2 X (number of parameters in the model). The model having the smallest value is the preferred model.^12^ AIC was applied to the Cox proportional hazards regression model to correct for potential bias in comparing prognostic systems with different numbers of parameters. Time‐dependent receiver operating characteristic (ROC) curves and estimated area under the curve (AUC) were used to compare prognostic abilities of the three classification systems. Time‐dependent ROC analysis is an extension of the ROC curve analysis and evaluates the power of discrimination of continuous indices for prognoses of time‐dependent disease.^13^ A predictive variable with a higher AUC indicates better discriminatory ability or prognostic accuracy. In addition, the discriminatory performance of the three classification systems was evaluated using Harrell's concordance index (C‐index).^14^ Harrell's C‐index is an extension of the AUC analysis to censored survival data.^14^ A larger C‐index value indicates a better ability to predict outcomes.

Data are presented as numbers of patients, proportions (%), median and interquartile range (IQR), or median and 95% confidence interval (CI), as indicated. *p *< 0.05 was considered statistically significant. All statistical analyses were conducted using the JMP14 software program (SAS Institute Japan Ltd.) and R ver.3.5.3 (R Foundation for Statistical Computing). The R package “stats,” “timeROC,” and “survival” were used for AIC analyses, time‐dependent ROC analyses, and C‐index analyses respectively.

## 3 RESULTS

### 3.1 Characteristics of the study cohort

The consort diagram for this study is shown in Figure S1. Between January 2000 and December 2017, 1245 patients with stage IV colorectal, appendiceal, and anal carcinoma were referred to our hospital. Excluding 11 patients with appendiceal cancer, six with anal cancer, three with neuroendocrine cell carcinoma, and three receiving chemotherapy for other concomitant advanced cancer, the final study population consisted of 1222 patients. The median follow‐up period for survivors was 38.4 months.

**FIGURE 1:**
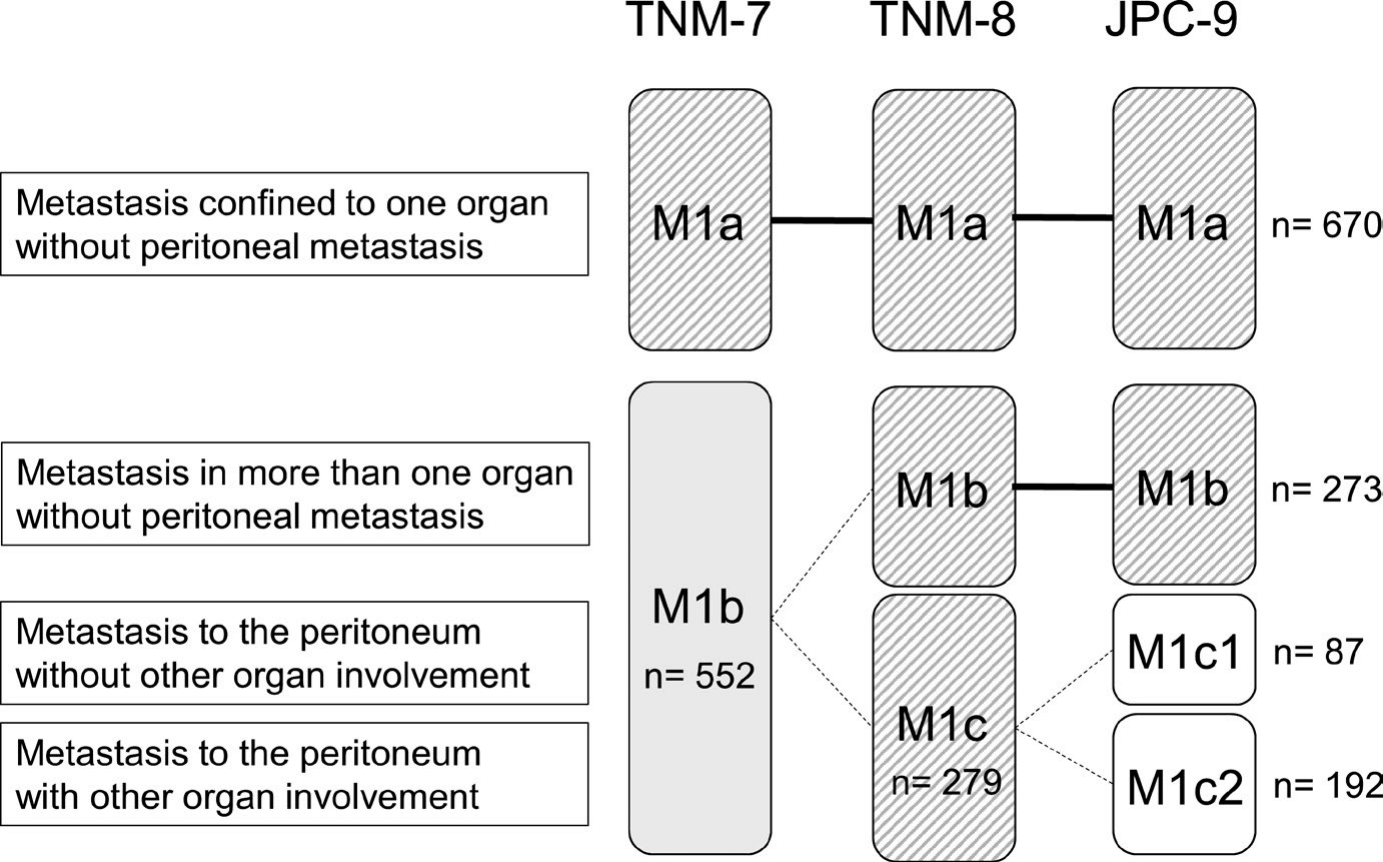
M categories in the TNM‐7, TNM‐8, and JPC‐9 classification systems. In TNM‐8, the M1b subcategory is divided into M1b and M1c. In JPC‐9, M1c is divided into M1c1 and M1c2

Patient characteristics are shown in Table 1. Median age was 61 years (IQR, 53–68 years), 693 patients (57%) were male, and 357 patients (29%) had right‐sided tumors. According to all three classification systems, 670 patients (55%) had M1a tumors (Table 1 and Figure 1). Moreover, 552 patients (45%) had M1b tumors using TNM‐7, and 273 (22%) and 279 (23%) had M1b and M1c tumors, respectively, using TNM‐8. According to JPC‐9, 87 (7%) patients had M1c1 tumors and 192 (16%) had M1c2 tumors (Figure 1).

**TABLE 1:** Clinical characteristics of patients

	Entire cohort *n *= 1222	Treatment year 2000–2007 *n *= 558	Treatment year 2008–2017 *n *= 664	*p* value
Age (years)	61 (IQR, 53–68)	60 (IQR, 53–67)	62 (IQR, 54–70)	0.0004
Gender				
Male	693 (57%)	327 (59%)	366 (55%)	0.221
Female	529 (43%)	231 (41%)	298 (45%)	
Primary tumor location				
Right‐sided	357 (29%)	164 (29%)	193 (29%)	0.901
Left‐sided	865 (71%)	394 (71%)	471 (71%)	
ECOG performance status				
PS0	704 (58%)	347 (62%)	357 (54%)	0.006
PS1	468 (38%)	197 (35%)	271 (41%)	
PS2	45 (4%)	12 (2%)	33 (5%)	
PS3, PS4	5 (0.4%)	2 (0.4%)	3 (0.5%)	
Tumor differentiation				
Differentiated	1095 (90%)	499 (89%)	596 (90%)	0.064
Poorly differentiated	81 (7%)	40 (7%)	41 (6%)	
Mucinous	26 (2%)	15 (2%)	11 (2%)	
Signet ring cell/undifferentiated	20 (1%)	4 (1%)	16 (2%)	
TNM−7				
M1a	670 (55%)	316 (57%)	354 (53%)	0.246
M1b	552 (45%)	242 (43%)	310 (47%)	
TNM−8				
M1a	670 (55%)	316 (57%)	354 (53%)	0.418
M1b	273 (22%)	116 (21%)	157 (24%)	
M1c	279 (23%)	126 (23%)	153 (23%)	
JPC−9				
M1a	670 (55%)	316 (57%)	354 (53%)	0.077
M1b	273 (22%)	116 (21%)	157 (24%)	
M1c1	87 (7%)	48 (9%)	39 (6%)	
M1c2	192 (16%)	78 (14%)	114 (17%)	
Type of surgery				
R0 resection	359 (29%)	189 (34%)	170 (26%)	<0.0001
Palliative primary tumor resection	400 (33%)	193 (35%)	207 (31%)	
Unresected	463 (38%)	176 (31%)	287 (43%)	
Type of chemotherapy (among patients who received systemic chemotherapy; *n *= 998)				
Cytotoxic agent therapy without targeted agents	554 (56%)	434 (91%)	120 (23%)	<0.0001
Targeted therapy	444 (44%)	45 (9%)	399 (77%)	

Data are presented as n (%). Numerical data are expressed as median (25%–75% interquartile range).

Abbreviations: CEA, carcinoembryonic antigen; IQR, interquartile range; JPC‐9, 9th edition of the Japanese Classification of colorectal, appendiceal, and anal carcinoma; TNM‐7, 7th edition of the tumor‐node‐metastasis classification; TNM‐8,8th edition of the tumor‐node‐metastasis classification.

Gender and primary tumor location did not differ between the 2000–2007 and 2008–2017 subgroups (*p *= 0.221 and *p *= 0.901, respectively), while age was significantly older and performance status was significantly worse in the 2008–2017 subgroup. Similarly, M category distributions did not significantly differ between the three classification systems (*p *= 0.246, *p *= 0.418, and *p *= 0.077 for TNM‐7, TNM‐8, and JPC‐9, respectively). Regarding the type of surgery, unresected cases increased from 31% in the 2000–2007 subgroup to 43% in the 2008–2017 subgroup (*p*<0.0001). Among patients who received systemic chemotherapy, proportions of those who received targeted therapy increased dramatically from 12% in the 2000–2007 subgroup to 77% in 2008–2017 subgroup (*p *< 0.0001). Five patients (0.9%) in the 2000–2007 subgroup and 11 patients (1.7%) in the 2008–2017 subgroup underwent conversion surgery (*p *= 0.24).

### 3.2 Long‐term outcomes

**FIGURE 2:**
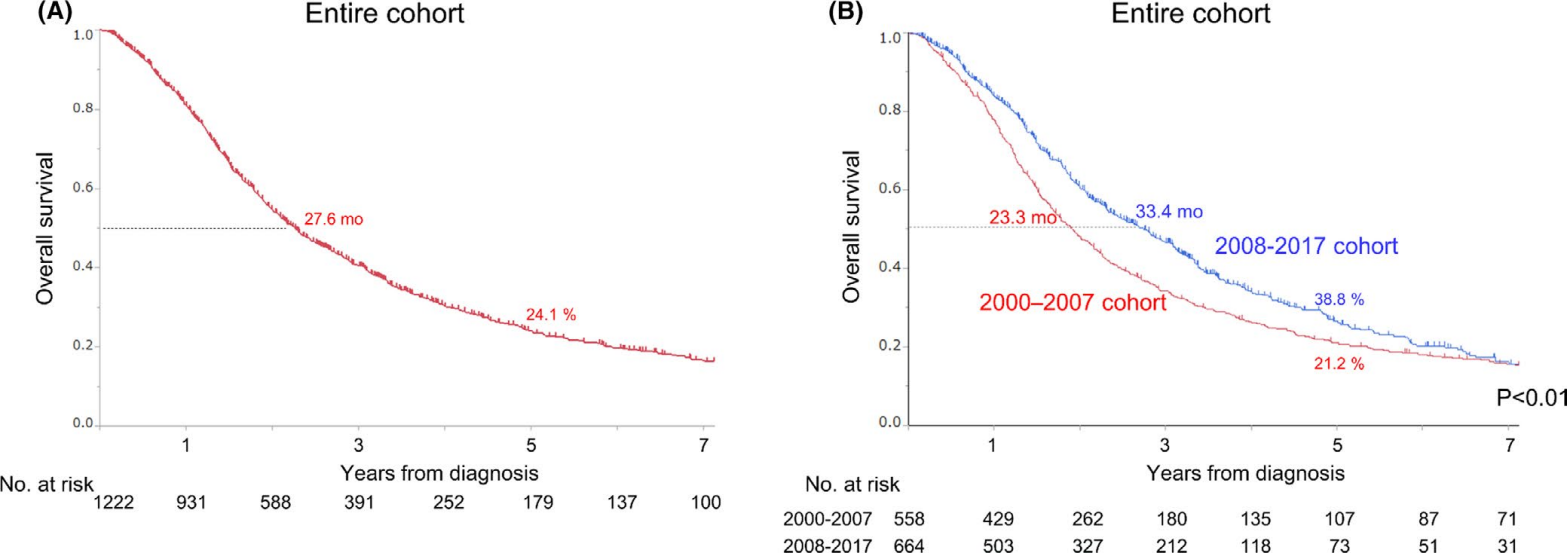
Overall survival curves for (A) stage IV colorectal cancer patients and (B) four groups stratified by initial treatment year

Figure 2A shows OS curves for the entire study cohort. The median follow‐up period was 38.4 months for surviving patients. Median survival time (MST) was 27.6 months and 5‐year OS rate was 24.1%. Figure 2B shows OS curves for the 2000–2007 and 2008–2017 subgroups. MST was 23.1 months for the 2000–2007 subgroup and 34.0 months for the 2008–2017 subgroup, demonstrating a significantly better OS for the 2008–2017 subgroup (Hazard ratio, 0.80; IQR, 0.70–0.92; *p *< 0.01) (data not shown).

**FIGURE 3:**
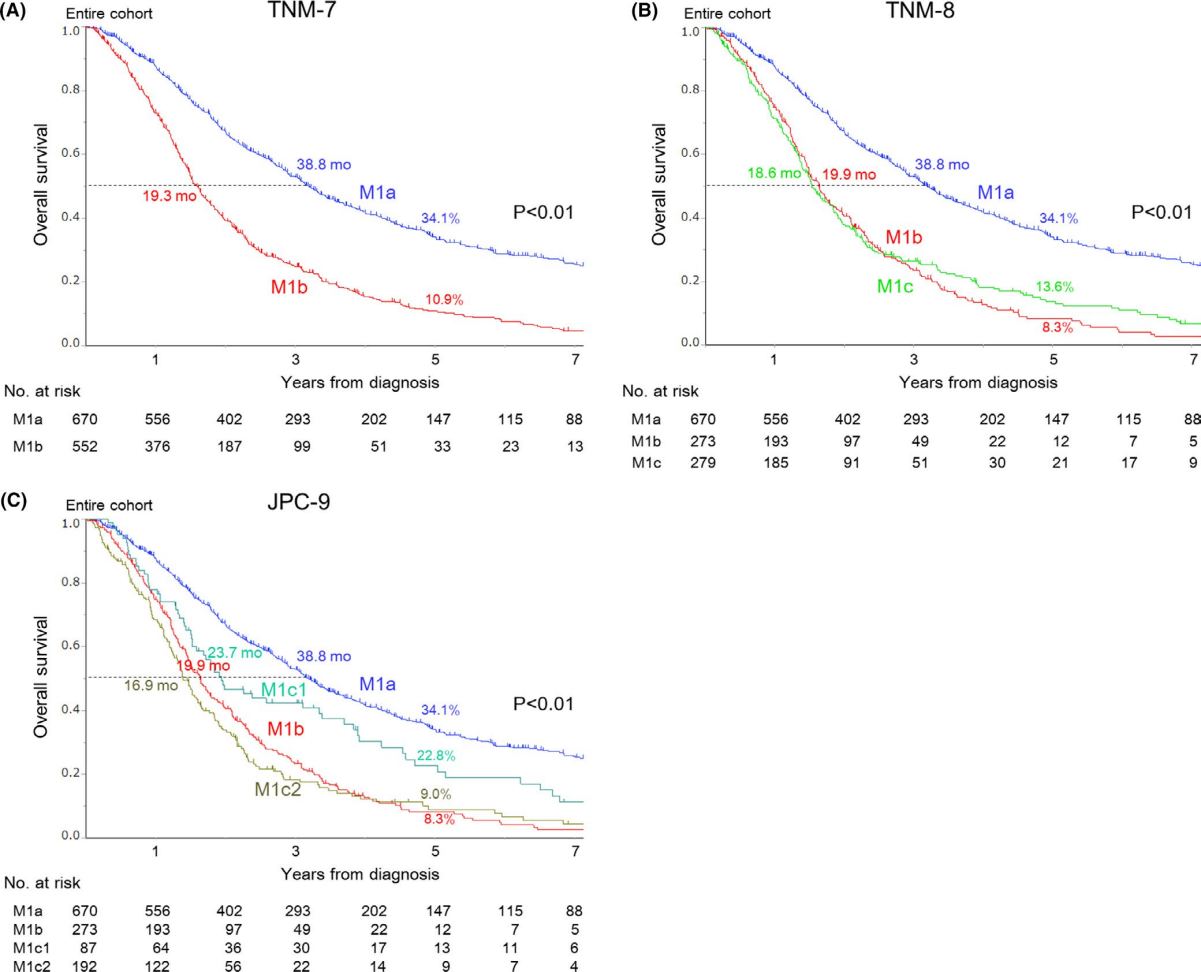
Overall survival curves for stage IV colorectal cancer patients according to (A) TNM‐7, (B) TNM‐8, and (C) JPC‐9

Figure 3 shows OS curves for stage IV colorectal cancer patients according to the three classification systems (Figure 3A: TNM‐7, Figure 3B: TNM‐8, Figure 3C: JPC‐9). In all three classification systems, OS of patients with M1a tumors was significantly longer than in patients with other M1 tumors (MST, 38.8 months; 5‐year OS, 34.1%). With TNM‐8, OS curves for patients with M1b and M1c tumors overlapped for the first three years, and the M1b curve eventually crossed below the M1c curve (Hazard ratio, 1.09; IQR, 0.90–1.31; *p *= 0.371) (data not shown). With JPC‐9, OS of patients with M1c1 tumors (MST, 23.7 months) was significantly longer than in patients with M1b (MST, 19.9 months) and M1c2 (MST, 16.9 months) tumors (M1c1 vs. M1b: Hazard ratio, 0.64; IQR, 0.48–0.85; *p *= 0.002; M1c1 vs. M1c2: Hazard ratio, 0.58; IQR, 0.43–0.77; *p *< 0.001) (data not shown).

### 3.3 Subgroup analyses of long‐term outcomes

**FIGURE 4:**
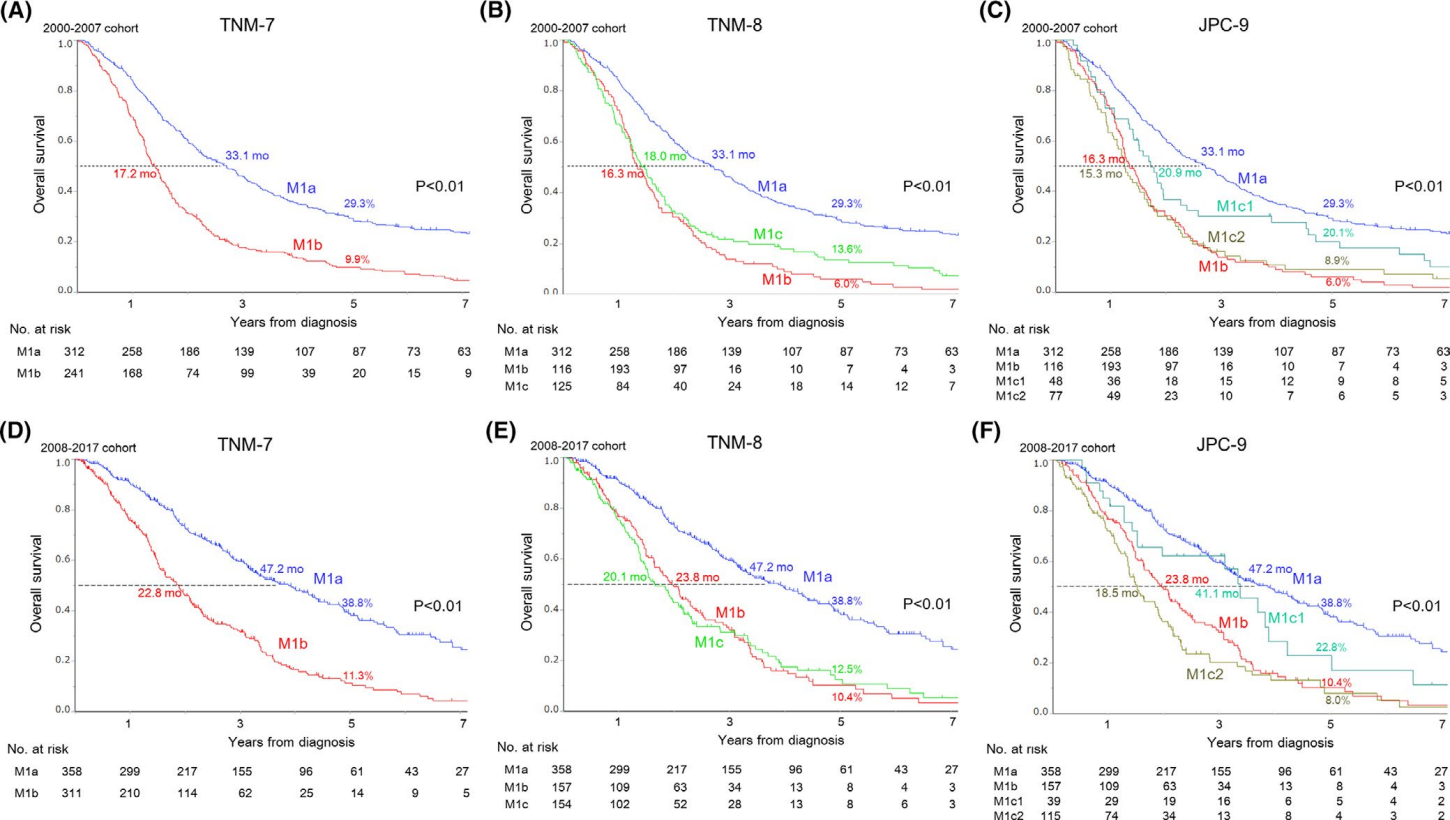
Subgroup analyses of overall survival curves stratified by treatment year for stage IV colorectal cancer patients according to the three classification systems (2000–2007 subgroup: (A) TNM‐7, (B) TNM‐8, and (C) JPC‐9; 2008–2017 subgroup: (d) TNM‐7, (E) TNM‐8, and (F) JPC‐9)

Subgroup analyses of OS stratified by initial treatment year revealed that OS of patients in each M subcategory for all classification systems was considerably longer in the 2008–2017 subgroup (e.g., M1a: MST of 33.1 months in 2000–2007 subgroup and 47.2 months in 2008–2017 subgroup) (Figure 4A vs. D, B vs. E, C vs. F). Notably, with TNM‐8, M1b, and M1c curves overlapped for the first three years in both subgroups, with the M1b curve eventually crossing below the M1c curve in the 2000–2007 subgroup but not in the 2008–2017 subgroup. With JPC‐9, patients with M1c1 tumors had better OS than those with M1b and M1c2 tumors in both subgroups (Figure 4C: MST 20.9 months vs. 16.3 months and 15.3 months; 4F: MST 41.1 months vs. 23.8 months and 18.5 months).

### 3.4 AIC values of the three classification systems

AIC values of each classification system are shown in Table 2. Analyses with the entire cohort revealed that the AIC value was lower for JPC‐9 compared to TNM‐7 and TNM‐8. Furthermore, subgroup analyses of AIC values by treatment year (2000–2007 and 2008–2017) were conducted (Table 2). In both subgroups, the AIC value was lower for JPC‐9 compared to TNM‐7 and TNM‐8.

**TABLE 2:** Akaike information criterion (AIC) and Harrell's concordance index (C‐index) for the three staging systems

	AIC		
	Entire cohort	Treatment year 2000–2007	Treatment year 2008–2017
TNM−7	10555.9	5013.4	4466.0
TNM−8	10585.5	5030.6	4478.7
JPC−9	10546.3	5011.2	4456.3
	Harrell's C‐index		
TNM−7	0.598 (SE = 0.009)	0.590 (SE = 0.012)	0.609 (SE = 0.013)
TNM−8	0.599 (SE = 0.009)	0.589 (SE = 0.013)	0.613 (SE = 0.014)
JPC−9	0.608 (SE = 0.009)	0.598 (SE = 0.012)	0.624 (SE = 0.014)

Abbreviations: JPC‐9, 9th edition of the Japanese Classification of colorectal, appendiceal, and anal carcinoma; SE, standard error; TNM‐7, 7th edition of the tumor‐node‐metastasis classification; TNM‐8, 8th edition of the tumor‐node‐metastasis classification.

### 3.5 Time‐dependent ROC analyses of OS

**FIGURE 5:**
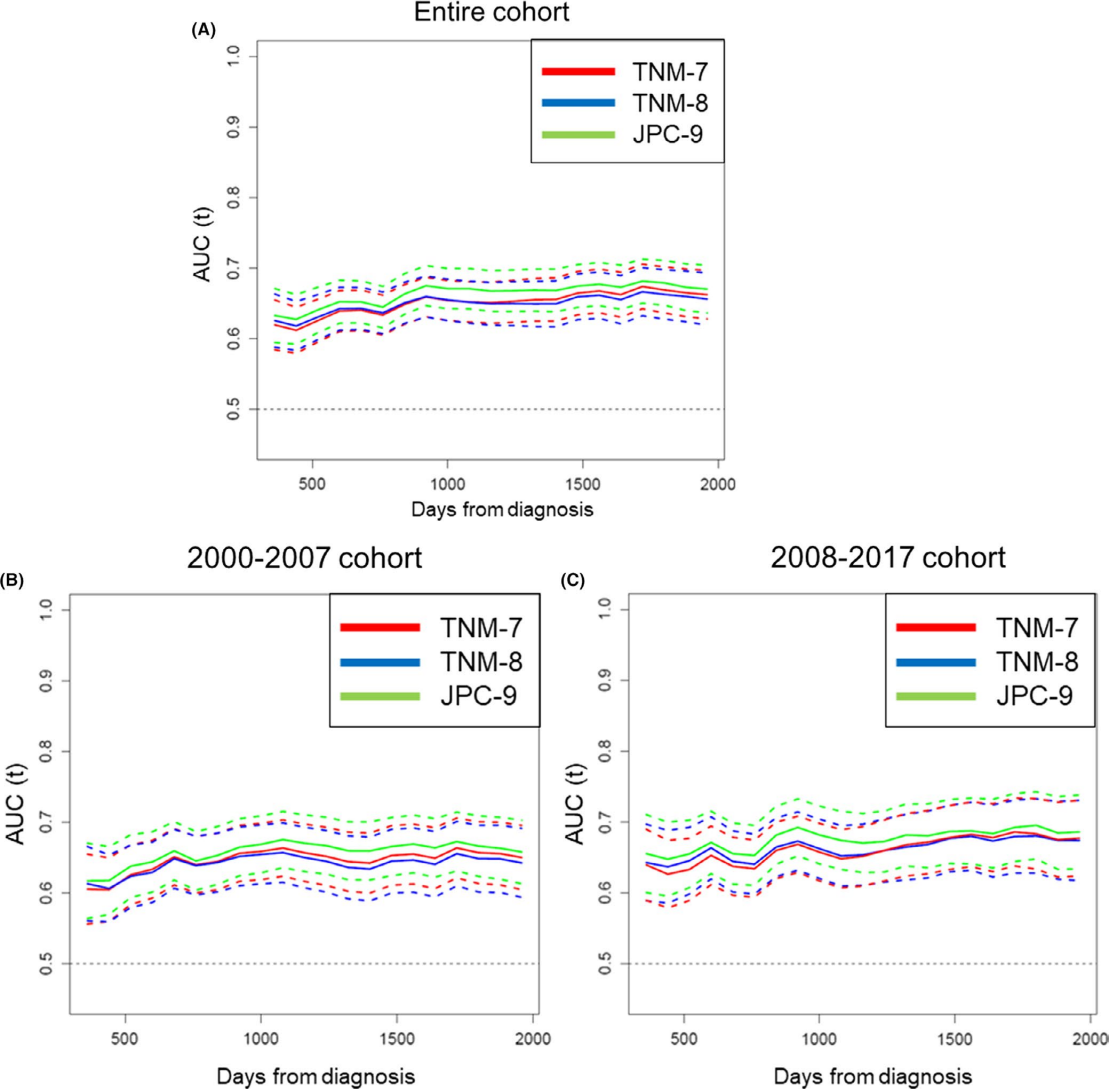
Time‐dependent receiver operating characteristic (ROC) curves with 95% confidence intervals for TNM‐7, TNM‐8, and JPC‐9. (A) Entire cohort, (B) 2000–2007 subgroup, and (C) 2008–2017 subgroup. AUC, area under the curve

Time‐dependent ROC curves were generated to compare sequential trends in discriminatory ability of the three classification systems for OS (Figure 5). The time‐dependent ROC curve for JPC‐9 was consistently superior to curves for TNM‐7 and TNM‐8 for all observation periods. Furthermore, subgroup analyses of time‐dependent ROC curves by treatment year revealed that the time‐dependent ROC curve for JPC‐9 was again superior to curves for TNM‐7 and TNM‐8 for all observation periods in both subgroups.

### 3.6 Harrell's C‐index of the three classification systems

Table 2 shows Harrell's C‐index of the three classification systems. Analyses with the entire cohort revealed that Harrell's C‐index was higher for JPC‐9 compared to TNM‐7 and TNM‐8 (Table 2). Furthermore, subgroup analyses by treatment year revealed that, in both subgroups, Harrell's C‐index was again higher for JPC‐9 compared to TNM‐7 and TNM‐8 (Table 2).

## 4 DISCUSSION

When considering a classification system to stage cancer patients, care must be taken in interpreting the results. Whereas two previous studies have shown that the prognosis of patients with peritoneal metastases is worse than that of patients with visceral metastases to one or more solid organs,^6^, ^7^ the patient populations of these studies were not uniformly stage IV colorectal cancer patients, but rather patients with unresectable metastatic colorectal cancer. Similarly, while a Japanese multi‐institutional retrospective study reported that the OS of patients with M1c1 tumors was significantly longer than that of patients with M1c2 or M1b tumors, 96% of patients subjected to analysis in that study had undergone resection of the primary tumor,^15^ implying that unresected cases comprised only 4% of the patient population and that not all stage IV colorectal cancer patients were included. In the present study, patients undergoing resection comprised 57% of the 2008–2017 subgroup. This is consistent with the annual rate of primary tumor resection in stage IV colorectal cancer patients of 57.4% in 2010, as reported using data from the National Cancer Institute's Surveillance, Epidemiology, and End Results CRC registry in the United States.^16^ Given that our data accurately represented real world stage IV colorectal cancer patient populations, we considered it feasible to compare the three classification systems for stage IV colorectal cancer in general. As a result, we found that JPC‐9, which subdivides peritoneal metastasis (M1c) based on the absence or presence of other organ involvement (M1c1 and M1c2, respectively), is superior to TNM‐7 and TNM‐8 for predicting OS in stage IV colorectal cancer patients.

The number of M1 subcategories varies across the three classification systems, with TNM‐7 having two subcategories (M1a and M1b), TNM‐8 having three subcategories (M1a, M1b, and M1c), and JPC‐9 having four subcategories (M1a, M1b, M1c1, and M1c2). The number of subcategories in the model is included when calculating AIC values, and in general, the greater the number of subcategories, the higher the AIC value. Nonetheless, despite having the highest number of subcategories, JPC‐9 had the lowest AIC of the three classification systems. These results suggest that dividing M1c into M1c1 and M1c2 contributes to improvements in model fit.

We also found that patients with M1c1 tumors had better OS than those with M1b and M1c2 tumors, regardless of treatment year. According to previous studies, when R0 resection of peritoneal metastasis was achieved, 5‐year OS rates ranged from 28.7%–34.1% among patients with M1c tumors.^17^, ^18^ Another study reported no significant difference in survival outcomes for patients with M1c tumors and patients with liver metastases who could achieve curative resection (5‐year OS rates: 32.1% and 33.3%, respectively),^19^ suggesting that long‐term outcomes of potentially resectable M1c tumors are comparable to those for other M1 tumors. In the present study, 4% (10/273) of M1b tumors and 7% (14/192) of M1c2 tumors achieved R0 resection, whereas 34% (30/87) of M1c1 tumors achieved R0 resection (data not shown). This could explain why patients with M1c1 tumors had better OS than those with M1b and M1c2 tumors. In other words, JPC‐9 allows for the extraction of patients with M1c1 tumors as a group with a favorable prognosis, accounting for about 30% of M1c patients. This may be the main reason why JPC‐9 has a better model fit and discriminatory ability compared to TNM‐7 and TNM‐8.

Since the classification system used must in principle be reliable regardless of the type of treatment or when the treatment was performed,^2^, ^3^ we also conducted subgroup analyses by dividing patients into two groups based on the period before and after introduction of targeted therapy in Japan. In this analysis, JPC‐9 was reliable both before and after the introduction of targeted therapy. Thus, JPC‐9 is valid also for patients with stage IV colorectal cancer who did not receive targeted therapy. On the other hand, TNM‐8 did not show a good model fit and its discriminatory ability improved only marginally relative to TNM‐7, and only in the 2008–2017 subgroup. This suggests that the update to the M category in TNM‐8 relative to TNM‐7 failed to consistently improve model fit and discriminatory ability over time.

This study has some limitations. First, because the study was retrospective in design, bias may exist. Second, although consecutive patients were enrolled, the study period was from 2000 to 2017. During this long period, treatment strategies including intensive chemotherapeutic regimens have changed significantly, as well as perioperative awareness of peritoneal metastasis. Thus, our study may not be fully reflective of current medical practice using newly developed treatment and diagnostic modalities. Third, a strategy unique to Japan (i.e., R0 resection of peritoneal metastasis from colorectal cancer^17^, ^18^, ^20^) was performed in some patients with limited peritoneal metastases, whereas in Western countries, cytoreductive surgery (peritoneal stripping surgery) with hyperthermic intraperitoneal chemotherapy (HIPEC) is more commonly performed. Since this strategy could affect the prognosis of patients with M1c tumors, our findings might not be generalizable to Western patient populations. That said, discussions on the extent of peritoneal resection for peritoneal metastases, such as ‘The extent of peritonectomy should vary according to the primary site. For colorectal peritoneal metastases, less extensive resection may be sufficient.’ Have begun in Western countries,^21^, ^22^ which might support a strategy unique to Japan. Fourth, since our data is based on treatments in Japan, there could be bias in evaluating the validity of the JPC‐9 using our data. However, there really is no major difference in treatment strategy between Japan and Western countries, as described above. And, basically, both the 7th and 8th editions of the TNM classification^2^, ^3^ mention that ‘a system of classification is needed that is applicable to all sites regardless of treatment’. Thus, regardless of the fact that our data were from Japan, it is reasonable to use the data to compare the three classification systems for stage IV colorectal cancer. Nonetheless, our findings warrant further consideration of M1c subcategorization and validation in a larger stage IV colorectal cancer patient population.

## 5 CONCLUSION

Updates to the M category from TNM‐7 to TNM‐8 failed to improve model fit and discriminatory ability. On the other hand, JPC‐9, which further divides M1c based on the presence or absence of other organ involvement, was superior to TNM‐7 and TNM‐8 for predicting OS in stage IV colorectal cancer patients. Our findings highlight the importance of updating staging classification systems regularly, particularly because new and important evidence accumulates even within the span of a few years. We anticipate that our results will serve as a reference for M1 category revision during the next update to the TNM classification system for malignant tumors.

## 6 ETHICAL APPROVAL STATEMENT

This retrospective study was approved by the Institutional Review Board (IRB) of the National Cancer Center Hospital (IRB code: 2015–320).

## CONFLICT OF INTEREST

The authors have no conflicts of interest to disclose.

## AUTHOR CONTRIBUTIONS

DS designed and coordinated the study, collected the data, analyzed and interpreted the data, and was responsible for writing the manuscript. NB participated in the design, collected the data, interpreted the data, and edited the manuscript. YN, TY, YY, KY, AT, and YK collected the data, performed the treatments, interpreted the data, and edited the manuscript. All authors read and approved the final manuscript.

## Supporting information

Fig S1

## References

[1] AJCC . AJCC cancer staging manual, 7th edn. New York, NY: Springer International Publishing; 2010.

[2] UICC . TNM classification of malignant tumours, 7th edn. New York, NY: Wiley‐Blackwell Ltd; 2009.

[3] UICC . TNM classification of malignant tumours, 8th edn. New York, NY: John Wiley & Sons Ltd; 2017.

[4] Segelman J , Granath F , Holm T , Machado M , Mahteme H , Martling A . Incidence, prevalence and risk factors for peritoneal carcinomatosis from colorectal cancer. Br J Surg. 2012;99:699‐705. 22287157 10.1002/bjs.8679

[5] Lemmens VE , Klaver YL , Verwaal VJ , Rutten HJ , Coebergh JW , de Hingh IH . Predictors and survival of synchronous peritoneal carcinomatosis of colorectal origin: a population‐based study. Int J Cancer. 2011;128:2717‐2725. 20715167 10.1002/ijc.25596

[6] Franko J , Shi Q , Meyers JP , et al. Prognosis of patients with peritoneal metastatic colorectal cancer given systemic therapy: an analysis of individual patient data from prospective randomised trials from the Analysis and Research in Cancers of the Digestive System (ARCAD) database. Lancet Oncol. 2016;17:1709‐1719. 27743922 10.1016/S1470-2045(16)30500-9

[7] Franko J , Shi Q , Goldman CD , et al. Treatment of colorectal peritoneal carcinomatosis with systemic chemotherapy: a pooled analysis of north central cancer treatment group phase III trials N9741 and N9841. J Clin Oncol. 2012;30:263‐267. 22162570 10.1200/JCO.2011.37.1039 PMC3269953

[8] AJCC . AJCC cancer staging manual, 8th edn. New York, NY: Springer International Publishing; 2017.

[9] JSCCR . Japanese Classification of Colorectal, Appendiceal, and Anal Carcinoma (9th edition). Tokyo: Kanehara & Co., Ltd.; 2018.

[10] Shida D , Kanemitsu Y , Hamaguchi T , Shimada Y . Introducing the eighth edition of the tumor‐node‐metastasis classification as relevant to colorectal cancer, anal cancer and appendiceal cancer: a comparison study with the seventh edition of the tumor‐node‐metastasis and the Japanese Classification of Colorectal, Appendiceal, and Anal Carcinoma. Jpn J Clin Oncol. 2019;49(4):321‐328. 30608547 10.1093/jjco/hyy198

[11] Shida D , Tanabe T , Boku N , et al. Prognostic value of primary tumor sidedness for unresectable stage IV colorectal cancer: a retrospective study. Ann Surg Oncol. 2019;26:1358‐1365. 30719633 10.1245/s10434-019-07209-x

[12] Kee K‐M , Wang J‐H , Lee C‐M , et al. Validation of clinical AJCC/UICC TNM staging system for hepatocellular carcinoma: analysis of 5,613 cases from a medical center in southern Taiwan. Int J Cancer. 2007;120:2650‐2655. 17304512 10.1002/ijc.22616

[13] Heagerty PJ , Lumley T , Pepe MS . Time‐dependent ROC curves for censored survival data and a diagnostic marker. Biometrics. 2000;56:337‐344. 10877287 10.1111/j.0006-341x.2000.00337.x

[14] Harrell FE Jr , Lee KL , Mark DB . Multivariable prognostic models: issues in developing models, evaluating assumptions and adequacy, and measuring and reducing errors. Stat Med. 1996;15:361‐387. 8668867 10.1002/(SICI)1097-0258(19960229)15:4<361::AID-SIM168>3.0.CO;2-4

[15] Tanaka T , Ozawa H , Nakagawa Y , Hirata A , Fujita S , Sugihara K . Verifying the M1c category of CRC: analysis of the data from a Japanese multi‐institutional database. Int J Colorectal Dis. 2020;35:125‐131. 31797096 10.1007/s00384-019-03408-w

[16] Hu C‐Y , Bailey CE , You YN , et al. Time trend analysis of primary tumor resection for stage IV colorectal cancer: less surgery, improved survival. JAMA Surg. 2015;150:245‐251. 25588105 10.1001/jamasurg.2014.2253

[17] Shida D , Tsukamoto S , Ochiai H , Kanemitsu Y . Long‐term outcomes after R0 resection of synchronous peritoneal metastasis from colorectal cancer without cytoreductive surgery or hyperthermic intraperitoneal chemotherapy. Ann Surg Oncol. 2018;25:173‐178. 29063295 10.1245/s10434-017-6133-7

[18] Shida D , Yoshida T , Tanabe T , Tsukamoto S , Ochiai H , Kanemitsu Y . Prognostic impact of R0 resection and targeted therapy for colorectal cancer with synchronous peritoneal metastasis. Ann Surg Oncol. 2018;25:1646‐1653. 29572704 10.1245/s10434-018-6436-3

[19] Cao CQ , Yan TD , Liauw W , Morris DL . Comparison of optimally resected hepatectomy and peritonectomy patients with colorectal cancer metastasis. J Surg Oncol. 2009;100:529‐533. 19697395 10.1002/jso.21369

[20] Shida D , Kobayashi H , Kameyama M , et al. Factors affecting R0 resection of colorectal cancer with synchronous peritoneal metastases: a multicenter prospective observational study by the Japanese Society for Cancer of the Colon and Rectum. Int J Clin Oncol. 2020;25:330‐337. 31677019 10.1007/s10147-019-01562-3

[21] Bhatt A , Glehen O . Extent of peritoneal resection for peritoneal metastases: looking beyond a complete cytoreduction. Ann Surg Oncol. 2020;27:1458‐1470. 31965374 10.1245/s10434-020-08208-z

[22] Bhatt A , Yonemura Y , Mehta S , et al. Target region resection in patients undergoing cytoreductive surgery for peritoneal metastases‐is it necessary in absence of visible disease? Eur J Surg Oncol. 2020;46:582‐589. 31757660 10.1016/j.ejso.2019.11.495

