# Supplementary figures and images for: Comparison of model fit and discriminatory ability of M category as defined by the 7th and 8th editions of the tumor‐node‐metastasis classification of colorectal cancer and the 9th edition of the Japanese classification
Source: Cancer Med. 2021 Sep 29;10(20):6937–46. doi: 10.1002/cam4.3972 (PMC8525077; doi:10.1002/cam4.3972)

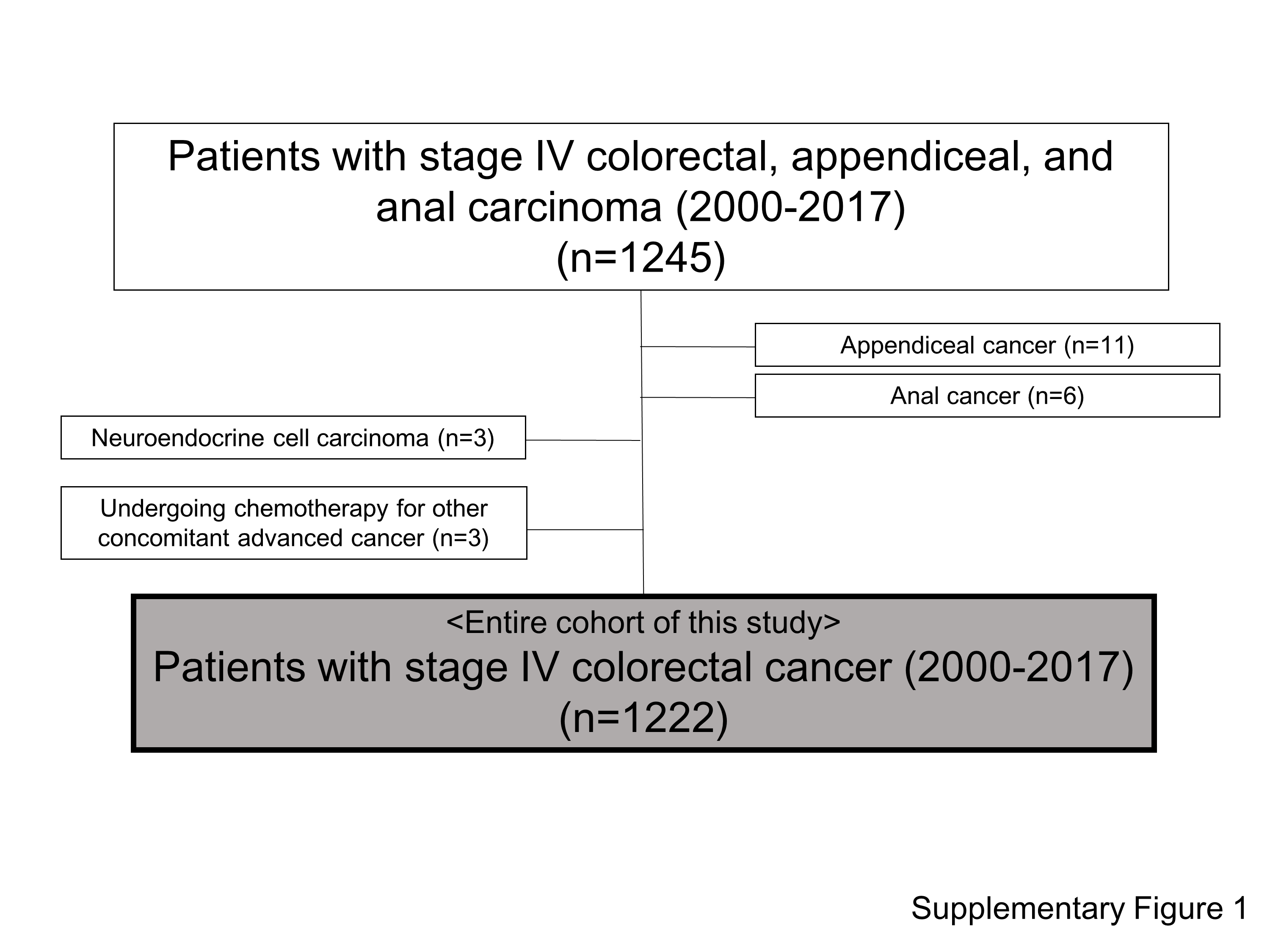

Supplement: Supplementary file 1 — Fig S1 [file CAM4-10-6937-s001.tif]
